# A Focused Review on Multiscale Characterization and Process–Structure–Property Linkages in Aerospace Die Forgings

**DOI:** 10.3390/ma19142953

**Published:** 2026-07-09

**Authors:** Lin Gao, Yu-Qing Zhang, Xiao Liu, Haitao Wang, Guozheng Quan

**Affiliations:** 1 Erzhong (Deyang) Heavy Equipment Co., Ltd., Deyang 618000, China; 2 Chongqing Key Laboratory of Advanced Mold Intelligent Manufacturing, School of Materials Science and Engineering, Chongqing University, Chongqing 400044, China

**Keywords:** aerospace die forging, multiscale analysis, correlative characterization, phase analysis, grain architecture, EBSD, titanium alloys, nickel-based superalloys, high-strength aluminum alloys, process–structure–property relationships

## Abstract

Aerospace die forgings are safety-critical structural products whose service performance is governed by coupled microstructural evolution across multiple length scales rather than by any single descriptor. This review critically synthesizes recent progress in multiscale characterization and process–structure–property analysis of aerospace die forgings, with emphasis on forged titanium alloys, wrought nickel-based superalloys, and high-strength aluminum alloys. A practical framework is first established by linking macroscale metal-flow integrity and defect control with mesoscale gradients, microscale grain-boundary and texture evolution, and nanoscale precipitation, segregation, and interface states. The principal characterization routes are then discussed, including X-ray diffraction, EBSD/3D-EBSD, TEM/STEM, atom probe tomography, tomography-based defect evaluation, and correlative workflows. The alloy-specific sections are organized around mechanisms and property consequences rather than isolated micrographs. Finally, the review discusses how multiscale descriptors can support crystal-plasticity, phase-field, cellular-automata, and ICME-oriented modeling, and identifies future priorities in three-dimensional characterization, quantitative descriptor extraction, uncertainty-aware modeling, environmental degradation assessment, and closed-loop process optimization. Overall, the performance of aerospace die forgings is shown to depend on coordinated control of phase stability, grain-boundary network evolution, precipitation state, defect population, and location-dependent heterogeneity across the full manufacturing route.

## 1. Introduction

Aerospace die forgings remain indispensable in modern airframe and aero-engine manufacturing despite the rapid development of additive and hybrid routes. Critical structural and rotating components, including landing-gear parts, compressor and fan disks, engine mounts, ring forgings, and heavily loaded wing or fuselage joints, are still predominantly forged because forging provides structural continuity, favorable load transfer, and certifiable damage tolerance. In this context, damage tolerance does not imply acceptance of defects; rather, it refers to quantitatively assessed flaw tolerance based on metrics such as ultrasonic indication size, fracture toughness (KIC/JIC), fatigue-crack-growth threshold (ΔKth), crack-growth rate (da/dN-ΔK), and fracture-mechanics-based critical flaw size. The final behavior of these forgings is therefore controlled by coupled evolution in phase constitution, grain structure, recrystallization, precipitation, defect inheritance, local heterogeneity, and service-induced degradation during billet breakdown, die filling, thermomechanical deformation, heat treatment, and service exposure [1,2,3,4,5,6,7,8,9,10,11,12].

Existing studies have established important alloy-specific foundations. In titanium alloys, recent reviews have clarified the roles of metastable β chemistry, α/β partitioning, β-grain evolution, recrystallization, and transformation-path dependence in forging-related microstructural control [1,2,3,4,5,13,14]. In wrought nickel-based superalloys, the literature has emphasized interactions among γ/γ′ microstructure, δ phase, carbides, precipitation-assisted recrystallization, and billet-state heterogeneity during disk-forging routes [6,7,8,9,10]. In high-strength aluminum alloys, studies on AA7050, AA7075, and Al-Li systems show that grain refinement, precipitation evolution, recrystallization, corrosion sensitivity, and anisotropy must be interpreted together when strength, toughness, ductility, and weight efficiency are optimized simultaneously [11,12,15,16,17,18,19,20,21,22,23,24,25,26,27,28].

At the same time, advances in characterization and modeling have made multiscale analysis increasingly tractable. EBSD and 3D-EBSD now provide quantitative access to grain morphology, boundary character, local misorientation, and parent-phase reconstruction [29,30,31,32,33,34,35,36]. TEM/STEM and atom probe tomography reveal dislocation substructures, interfaces, nanoscale precipitates, clustering, and segregation [37,38]. In parallel, microstructure-sensitive crystal plasticity, phase-field methods, and coupled multiscale simulations provide the means to translate measured descriptors into predictive process–structure–property frameworks [39,40,41,42,43,44,45,46,47,48,49].

In this review, the multiscale approach is defined as a correlative workflow rather than a catalog of instruments. Process variables are first mapped to spatially resolved descriptors at macro-, meso-, micro-, sub-micro-, and nano/atomic levels; these descriptors are then registered across the same component locations or representative material volumes; finally, they are used as inputs, calibration targets, or validation evidence for mechanics-based and data-enabled models. Thus, multiscale characterization requires scale linking, descriptor transfer, and uncertainty control, while multiscale modeling requires the propagation of information from forging history to local structure and ultimately to property response.

**Figure 1:**
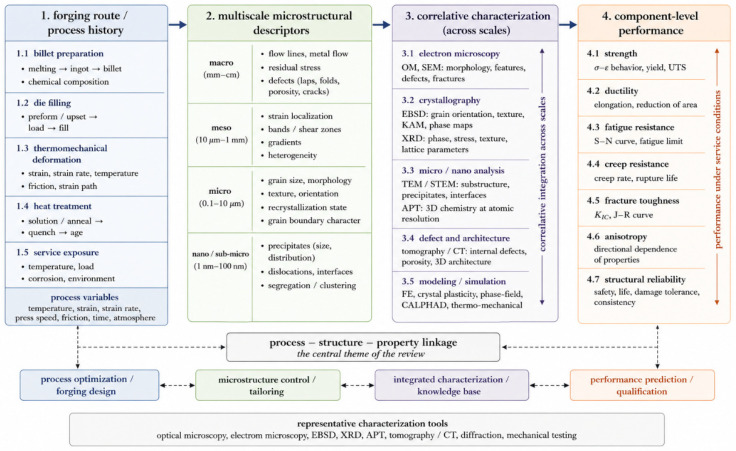
Author-created schematic. Multiscale review framework for aerospace die forgings, showing how forging history, hierarchical microstructural descriptors, correlative characterization, and component-level performance are linked within a process–structure–property framework.

Despite this progress, the literature remains fragmented. Most published reviews are organized either by alloy family or by a single mechanism such as phase transformation, recrystallization, precipitation, or damage. In engineering practice, however, aerospace forgings must be evaluated through interconnected questions: which phases are present and how stable they remain within a given forging window; how grain size, morphology, texture, and recrystallized fraction evolve; how precipitates, dislocations, interfaces, surface condition, and defects are distributed locally; and how such observations can be transformed into predictive process–structure–property relationships [13,14,29,30,31,32,33,34,35,36,37,38,39,40,41,42,43,44,45,46,47,48,49,50,51,52,53,54,55,56,57,58,59,60,61,62,63,64,65,66,67,68,69,70,71,72,73,74,75,76,77,78,79,80,81,82,83,84]. Figure 1 summarizes this integrated logic by linking forging history, hierarchical descriptors, correlative characterization, and component-level response.

Accordingly, the present review adopts multiscale analysis as its organizing principle. It focuses on phase analysis, grain and texture characterization, recrystallization behavior, precipitate and interface evolution, surface/environmental degradation, defect assessment, and predictive modeling in forged aerospace alloys. Particular attention is given to titanium alloys, wrought nickel-based superalloys, and high-strength aluminum alloys because these three groups represent the principal material systems used in aerospace die forgings and collectively cover the dominant multiscale phenomena encountered in manufacturing and service.

## 2. Multiscale Analysis Framework for Aerospace Die Forgings

### 2.1. Hierarchical Structure and Key Analysis Targets

Aerospace die forgings should be understood as hierarchical structural materials whose service reliability is established progressively across multiple scales. At the macroscale, the key concerns are complete die filling, metal-flow continuity, dimensional accuracy, and mitigation of feedstock-inherited defects such as segregation, porosity, or flow discontinuities. At the mesoscale, strain path, die constraint, friction, and thermal gradients produce location-dependent heterogeneity, including surface-core differences, localized deformation bands, and nonuniform recrystallization. At the microscale, decisive descriptors include grain size distribution, grain morphology, crystallographic orientation, boundary character, and recrystallized fraction. At the sub-micro and nano/atomic scales, dislocation structures, interfaces, precipitate chemistry, and segregation states directly affect strengthening, damage initiation, and thermal stability. In this review, the term “scale” includes linear dimensions, areal descriptors, and volumetric descriptors because the relevant observation domain is determined by both feature size and measurement dimensionality.

**Figure 2:**
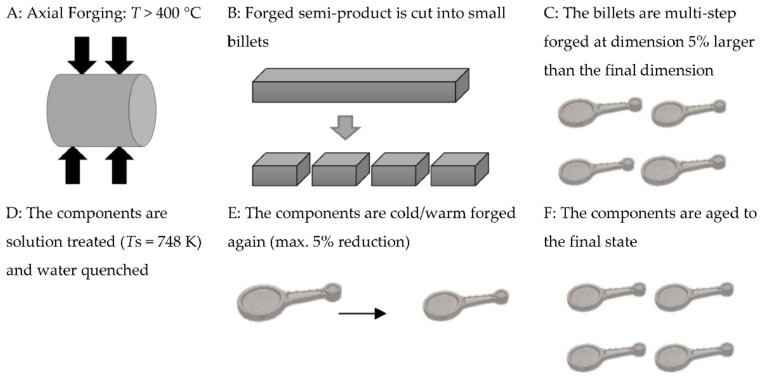
Representative forging-process route for an aerospace aluminum alloy, illustrating how bulk deformation history and section conversion establish the macro-scale basis for subsequent multiscale microstructural analysis [11]. Adapted from Ref. [11] under CC BY 4.0.

A useful multiscale framework should therefore be built around analysis targets rather than instruments alone. The principal targets include phase fraction and topology, parent-phase reconstruction, grain size and grain-shape tensors, low-angle and high-angle grain-boundary density, local orientation gradients, recrystallized and unrecrystallized domains, precipitate type and number density, pore or inclusion distribution, surface topography, and the directional dependence of mechanical response. Organizing the discussion in this way allows phase analysis, grain analysis, precipitation analysis, surface analysis, and defect assessment to be integrated into a single process–structure–property narrative. A representative forging-process route is shown in Figure 2 to emphasize how macroscopic deformation history provides the starting point for later multiscale descriptors.

Grain size and nanoscale structural units have direct mechanical significance. At the micrometer scale, grain refinement usually increases yield strength through a Hall-Petch-type relation, while the grain-size distribution and boundary character also influence fatigue-crack initiation, dwell sensitivity, and fracture-path tortuosity. At the nanometer scale, precipitate radius, number density, interparticle spacing, and solute segregation control precipitation strengthening, local strain partitioning, and thermal stability. Consequently, mechanical and structural properties should be related to both micrometer-scale grain architecture and nanometer-scale phase/precipitate descriptors rather than to either level alone.

For surface-affected zones, multiscale analysis should also include scale-dependent topography and surface-functionality descriptors. Established methods include Fourier-based spectral analysis, wavelet decomposition, band-pass filtering, morphological filtering, and fractal or geometric decomposition, which separate roughness, waviness, texture directionality, and larger-form features across different observation windows. These methods are relevant to forged aerospace components because die contact, surface finishing, shot peening, oxidation, corrosion, and coating adhesion all depend on surface features that may be linear, areal, or volumetric in nature [85].

### 2.2. Core Characterization Routes Across Scales

No single characterization technique can describe the full structural complexity of aerospace die forgings. Multiscale understanding instead depends on the coordinated use of complementary methods. X-ray diffraction is useful for phase screening and residual-stress assessment; EBSD is indispensable for quantifying grain orientation, boundary misorientation, recrystallized fraction, local misorientation, and texture evolution; TEM and STEM resolve dislocation substructures, interfaces, and nanoscale precipitates; atom probe tomography provides near-atomic chemical information on clustering, partitioning, and segregation; and tomography-based techniques identify internal soundness, pore distribution, and inherited damage. These methods are most powerful when applied as a correlative sequence rather than as isolated observations.

**Figure 3:**
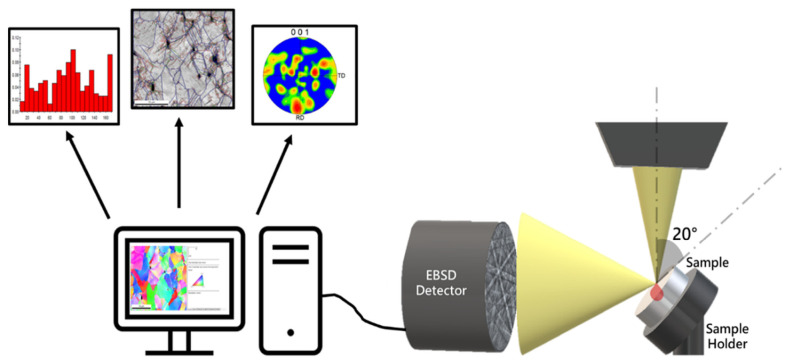
Schematic of the EBSD setup and data-acquisition logic used for grain, orientation, and boundary analysis in forged metallic materials [29]. Adapted from Ref. [29] under CC BY 4.0.

Correlative analysis is more informative than an instrument-by-instrument inventory because the central question is whether the selected methods can bridge forging variables, local structural descriptors, and performance consequences. In aerospace die forgings, the most robust studies combine bulk methods, spatially resolved orientation mapping, nanoscale characterization, and local mechanical assessment so that phase stability, grain-boundary evolution, precipitate chemistry, and component-level heterogeneity can be interpreted within the same local volume. Figure 3 illustrates the EBSD setup and data products that support grain- and orientation-based analysis, while Table 1 summarizes representative targets, methods, and outputs at different scales.

### 2.3. Correlative Interpretation Logic and Section Integration

The framework adopted in this review uses multiscale analysis as the primary organizing thread and treats alloy-specific discussions as material case studies within that unified structure. Titanium alloys mainly emphasize phase partitioning, β-grain reconstruction, and deformation-induced microtexture inheritance. Nickel-based superalloys highlight the coupling among precipitation state, recrystallization pathway, grain-boundary evolution, and billet heterogeneity. High-strength aluminum alloys provide clear examples of how precipitation, recrystallization, texture, anisotropy, and corrosion sensitivity remain interconnected. Although the dominant mechanisms differ across systems, all three alloy classes demonstrate the same principle: forging behavior must be interpreted through coordinated control of phase stability, grain evolution, interfacial state, local heterogeneity, and surface condition rather than through a single average descriptor.

**Figure 4:**
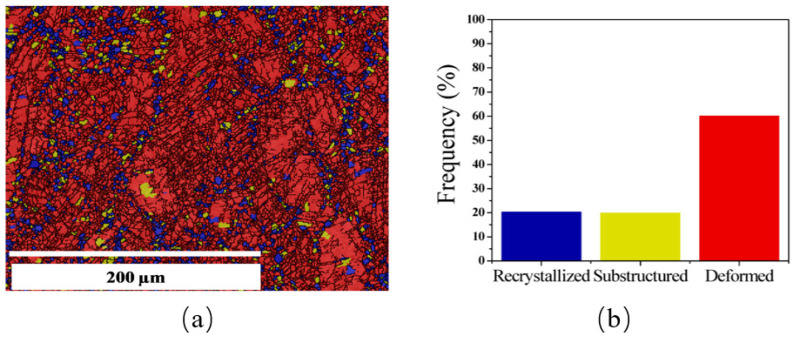
Representative EBSD-based recrystallized-fraction map showing the transition among recrystallized, substructured, and deformed grain populations after thermomechanical processing [76]. Adapted from Ref. [76] under CC BY 4.0. (**a**) EBSD recrystallization-state map; (**b**) frequency distribution of recrystallized, substructured, and deformed grain populations.

This correlative logic is especially valuable for review writing because it allows alloy-specific evidence to be compared on a common basis. In the present section, an EBSD-based recrystallized-fraction map is used as a representative example of how local grain-state statistics can be connected to thermomechanical history and later interpretation across scales. Figure 4 therefore serves as a bridge between the general framework developed in Section 2 and the alloy-specific analyses presented in the following sections.

## 3. Titanium Alloy Die Forgings

### 3.1. Phase Constitution and Forging-Window Sensitivity

Titanium alloy die forgings provide a clear demonstration of why multiscale analysis is essential. Their final performance is highly sensitive to the thermomechanical window selected relative to the β transus; therefore, the forging schedule controls not only final geometry but also phase stability, grain-boundary migration, recovery and recrystallization behavior, and the morphology of transformation products formed during cooling and aging [1,2,3,4,5,13,14,54,55,56,57,58,59,60,61,62,63,64,65,66,67,68,69,70].

For near-α and α + β titanium alloys, the central issues include globularization of lamellar α, fragmentation of colony structures, transformed-β morphology, and the retention or weakening of deformation-induced texture. In near-β and metastable β titanium alloys, by contrast, the dominant variables are prior β-grain topology, the competition between dynamic recovery and recrystallization, and the later precipitation of α from the β matrix during post-forging heat treatment. The key point is that titanium forgings cannot be classified adequately by alloy family alone; they must be interpreted through the sensitivity of phase constitution and grain architecture to the forging window itself.

**Figure 5:**
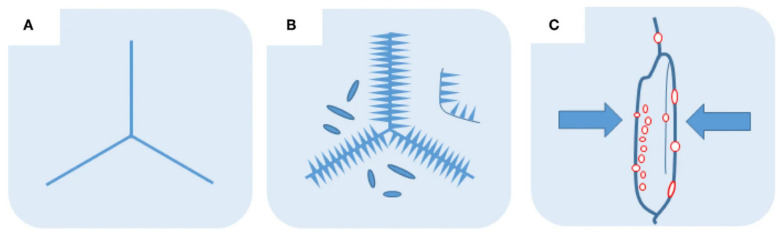
Schematic of phase-state evolution in a near-β titanium alloy during solution treatment, aging, and isothermal compression, illustrating how the forging and heat-treatment window controls subsequent microstructural descriptors. Adapted from Ref. [55] under CC BY 4.0. (**A**) solution-treated near-β matrix with prior β grain boundaries; (**B**) aging-induced α precipitation along boundaries and within grains; (**C**) isothermal compression, where arrows indicate the applied compressive loading and red circles mark stress-induced α-related features.

A physically meaningful description of titanium forging should therefore follow the sequence from forging temperature and strain path to parent β-state evolution and, finally, to transformed α/β microstructure after cooling or aging. Studies on Ti-7333, Ti-10V-2Fe-3Al, TB18, and related near-β systems show that deformation temperature, strain rate, pass schedule, and sub-transus versus near-transus processing alter not only grain size but also parent-state inheritance, recrystallization mode, and variant selection during subsequent phase transformation. Figure 5 summarizes this forging-window sensitivity through a representative phase-state evolution schematic.

For Ti-10V-2Fe-3Al and similar near-β titanium alloys, the most useful state for high-quality aerospace forgings is generally a controlled α + β microstructure obtained by deformation/solution treatment in a suitable near-β or sub-transus window, followed by aging to precipitate fine secondary α within a beta matrix. A fully β-dominated state provides good hot workability but insufficient final strengthening if it is not aged, whereas excessive or coarse α can reduce toughness and damage tolerance. Phase transformation therefore directly changes strength, ductility, fatigue resistance, dwell sensitivity, and anisotropy through the α/β fraction, α morphology, variant selection, and retained/metastable β stability.

### 3.2. β Reconstruction, Recrystallization, and Microtexture

Recent titanium-forging research has moved beyond conventional optical or SEM description and increasingly relies on EBSD-based parent β reconstruction, recrystallization mapping, and microtexture analysis. This shift is important for near-β titanium alloys because nominally similar forged components may develop different transformed-α morphologies after heat treatment if their prior β network, boundary mobility, and local crystallographic neighborhoods differ.

The literature also shows that recrystallization in titanium forgings is commonly heterogeneous rather than spatially uniform. In Ti-7333, recrystallized grains were reported to inherit orientations from the deformed matrix during hot rolling, whereas in Ti-10V-2Fe-3Al, β recrystallization in the α + β regime was described as non-homogeneous among prior β grains and accompanied by enriched sub-grains, chain-like or sporadic recrystallized β grains, and wave-shaped β grain boundaries [52,56]. These observations demonstrate that local β reconstruction and recrystallization behavior directly condition the later transformation path rather than merely refining the structure in a generic sense.

**Figure 6:**
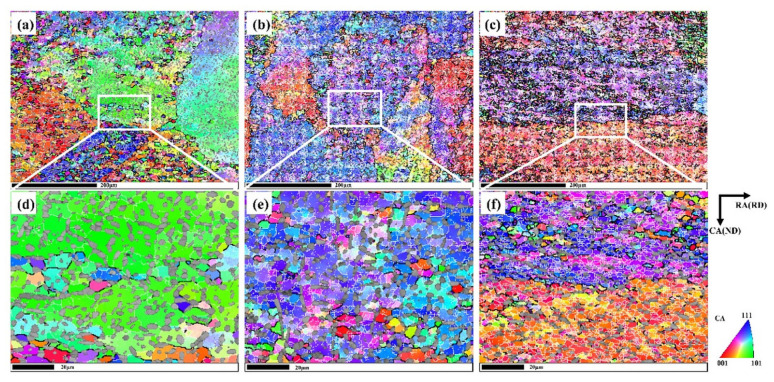
Orientation distribution maps of the β phase at different strain rates, showing heterogeneous β recrystallization, sub-grain development, and deformation-induced microtexture evolution in a near-β titanium alloy. Adapted from Ref. [56] under CC BY 4.0. (**a**) overall β-phase orientation map under deformation condition I; (**b**) overall β-phase orientation map under deformation condition II; (**c**) overall β-phase orientation map under deformation condition III; (**d**) enlarged view of the boxed region in (**a**); (**e**) enlarged view of the boxed region in (**b**); (**f**) enlarged view of the boxed region in (**c**).

The most informative titanium studies therefore connect at least three levels simultaneously: the imposed thermomechanical history, the reconstructed phase or grain architecture, and the implications for local deformation compatibility or property scatter. This linkage distinguishes genuine multiscale analysis from descriptive micrograph collection. Figure 6 provides a representative orientation-map example showing how β-phase texture and recrystallization heterogeneity evolve under different deformation conditions.

### 3.3. Property Linkage and Review Implications

The titanium literature makes it clear that phase analysis and grain analysis cannot be separated when aerospace die forgings are interpreted mechanistically. β-grain refinement without phase-sensitive interpretation is often insufficient to explain the morphology, distribution, and orientation relationship of the subsequently formed α phase. Conversely, discussion of α precipitation or transformed microstructure without boundary character, local misorientation, and parent β information rarely explains the observed anisotropy or scatter in mechanical response.

From a performance standpoint, this integration matters because critical damage modes in aerospace titanium forgings are often linked to local rather than average microstructural states. Microtexture inheritance, recrystallization heterogeneity, Burgers-orientation-related transformation behavior, and local misorientation accumulation all influence strain partitioning, crack nucleation propensity, dwell sensitivity, and property anisotropy. Recent work therefore increasingly emphasizes local misorientation metrics, grain-boundary statistics, β reconstruction, and microstructure-sensitive interpretation instead of relying on average grain-size descriptors alone.

**Figure 7:**
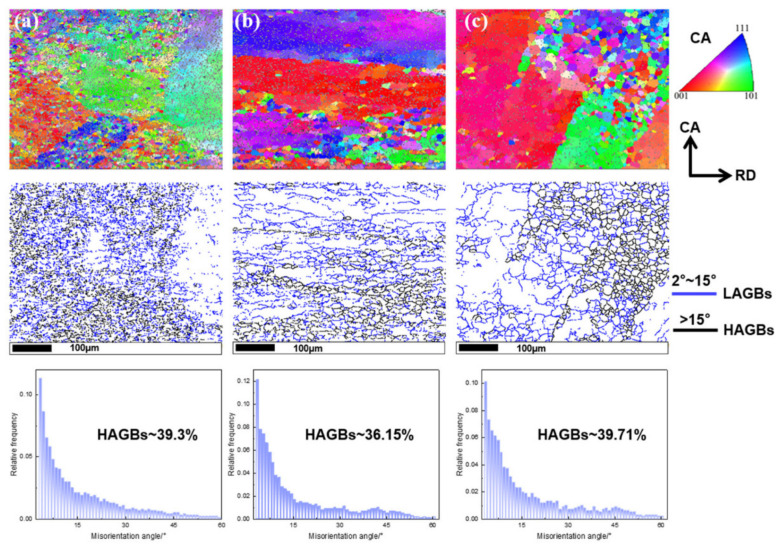
Grain-boundary misorientation distributions and corresponding IPF maps of deformed β grains, illustrating how boundary-state heterogeneity can be linked to microtexture evolution and performance-relevant interpretation in titanium forgings [56]. Adapted from Ref. [56] under CC BY 4.0. (**a**) deformation condition I; (**b**) deformation condition II; (**c**) deformation condition III. Each column contains the IPF map, boundary map, and misorientation distribution for the corresponding condition.

Taken together, these studies show that titanium die forgings are best understood as integrated process–structure–property systems. The dominant interpretive chain is forging path > phase and grain inheritance > local heterogeneity > property consequence. Figure 7 highlights this logic through representative grain-boundary-misorientation and IPF evidence that links boundary-state evolution to microtexture development and performance-relevant heterogeneity.

## 4. Wrought Nickel-Based Superalloy Die Forgings

### 4.1. Precipitation-Controlled Phase Stability

Wrought nickel-based superalloy die forgings provide a particularly strong example of multiscale interpretation because their microstructural evolution is governed not by grain size alone but by the continuous interaction between precipitation state and recrystallization behavior. In forged aero-engine materials, the γ matrix and γ′ precipitates define the basic strengthening framework, while δ phase, carbides, borides, and other secondary constituents regulate grain-boundary mobility, local deformation compatibility, and grain-growth resistance during billet conversion and subsequent disk forging [6,7,8,9,10,71,72,73,74,75,76,77,78,79,80,81,82,83,84].

Accordingly, phase stability in forged superalloys must be interpreted together with the thermomechanical path and billet-state heterogeneity. The relevant question is not only whether γ′ is present, but how the size, distribution, dissolution, and reprecipitation of γ′ and related phases alter boundary migration and the accessibility of different recrystallization pathways under sub-solvus or near-solvus processing conditions. To avoid potential copyright restrictions associated with the previous schematic, the figure has been removed, and the mechanism is described textually in the revised manuscript.

### 4.2. Recrystallization, Grain-Boundary Character, and Billet Heterogeneity

Recent literature on wrought nickel-based superalloy forging highlights three recurring themes. First, billet-state heterogeneity matters greatly. Billet-conversion studies on AD730 have shown that intermediate stock can contain heterogeneous mixtures of coarse and fine recrystallized grains, unrecrystallized regions, and nonuniform γ′ precipitation; consequently, different regions of the same billet may recrystallize through different operative mechanisms [75].

Second, the classical distinction between discontinuous and continuous dynamic recrystallization becomes more informative when it is interpreted together with precipitation behavior. In AD730 and related systems, precipitates may stimulate discontinuous dynamic recrystallization by providing nucleation sites, impede recrystallized grain growth through pinning, or promote gradual continuous recrystallization, depending on local phase state and deformation conditions [73,75,77,79,80].

**Figure 8:**
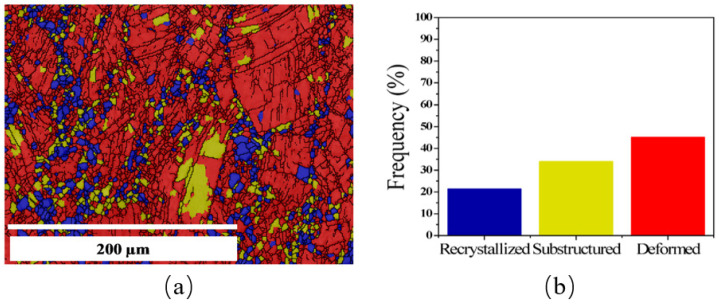
Recrystallized-fraction map for a δ-processed Ni-based superalloy after deformation, distinguishing recrystallized, substructured, and deformed grain populations and illustrating the spatial heterogeneity of restoration behavior. Adapted from Ref. [76] under CC BY 4.0. (**a**) EBSD recrystallization-state map; (**b**) frequency distribution of recrystallized, substructured, and deformed grain populations.

**Figure 9:**
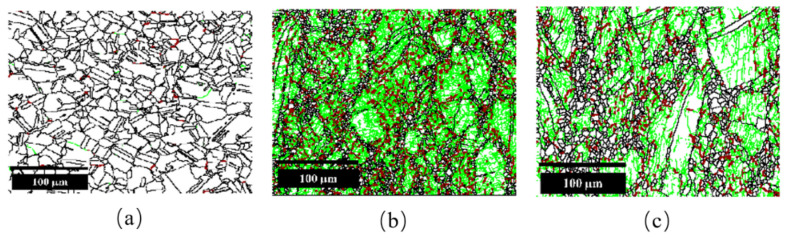
EBSD grain-boundary map showing the distribution of low-angle and high-angle boundaries after deformation, highlighting the sensitivity of boundary-state evolution to temperature and strain-rate history. Adapted from Ref. [76] under CC BY 4.0. (**a**) low-angle/high-angle boundary distribution under deformation condition I; (**b**) boundary distribution under deformation condition II; (**c**) boundary distribution under deformation condition III.

Third, grain-boundary character is not merely a descriptive output but an active variable in phase evolution and microstructural stability. In the δ-processed Ni-based superalloy studied by Paramo-Kanetas et al., recrystallized-fraction maps distinguished recrystallized, substructured, and deformed grain populations, while the fraction of low-angle and high-angle boundaries changed strongly with deformation condition [76]. Figure 8 and Figure 9 illustrate this coupling between recrystallization fraction, boundary-state evolution, and processing history.

The experimentally observed heterogeneity illustrated in Figure 8 has two different implications. Uncontrolled heterogeneity is generally detrimental because coarse/fine grain mixtures, uneven γ′ or δ distributions, and unrecrystallized bands create local differences in yield strength, creep resistance, fatigue-crack initiation resistance, and crack-growth path. These differences increase property scatter and reduce certification confidence. However, intentionally designed heterogeneity may be useful when it is spatially controlled, for example in dual-microstructure disks where a fine-grained bore can improve fatigue resistance and a coarser rim can improve creep resistance. Thus, heterogeneity is advantageous only when it is deliberately designed, quantitatively characterized, and linked to location-specific service requirements; otherwise, it should be treated as a risk factor in aerospace forgings.

### 4.3. Multiscale Review Implications for Disk-Forging Performance

The superalloy literature therefore makes a full cascade explicit: phase constitution > precipitate evolution > recrystallization pathway > boundary-state development > performance sensitivity. This interpretation is directly relevant to aero-engine applications because sub-solvus versus super-solvus processing, local grain-boundary-character distribution, and persistence of billet heterogeneity all influence the microstructural uniformity required for creep- and fatigue-sensitive service.

For disk-forging materials, the central implication is that property scatter should be viewed as the consequence of local differences in phase stability, boundary pinning, and recrystallization kinetics rather than as random variability. A rigorous review of forged nickel-based superalloys should therefore integrate phase constitution, grain-boundary analysis, billet history, and thermomechanical path within the same interpretive framework.

## 5. High-Strength Aluminum Die Forgings

### 5.1. Precipitation and Phase Decomposition Pathways

High-strength aluminum die forgings, especially AA7050, AA7075, and related Al-Zn-Mg-Cu alloys, provide a complementary multiscale case in which precipitation, texture, corrosion sensitivity, and anisotropy are tightly coupled. Compared with titanium alloys, where parent-phase reconstruction is often central, or superalloys, where precipitation strongly mediates recrystallization kinetics, forged high-strength aluminum alloys are governed by the joint evolution of matrix precipitates, grain-boundary precipitation, dispersoids, and partially recrystallized grain structures [11,12,15,16,17,18,19,20,21,22,23,24,25,26,27,28].

The key scientific issue is therefore not simply attainment of high strength but redistribution of strengthening phases and deformation substructure across grains, boundaries, and section directions during forging, solution treatment, and aging. In AA7050 forging, intermediate deformation before aging was shown to modify both the final property level and the underlying precipitation state and grain morphology, thereby improving fracture-toughness stability without sacrificing tensile properties [11].

### 5.2. Grain Architecture, EBSD, and Anisotropy Assessment

Recent studies on AA7050 and AA7075 show that EBSD, TEM, and related correlative methods are especially valuable when used together. Their main strength is that they resolve not only grain size and morphology but also local orientation gradients, low-angle and high-angle boundary evolution, geometrically necessary dislocation accumulation, and direction-dependent recrystallization under hot-forging conditions.

**Figure 10:**
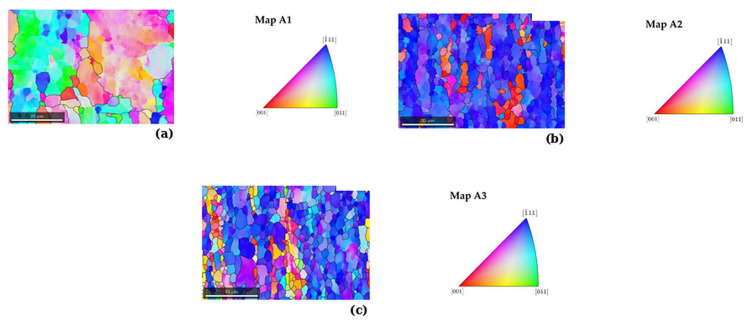
EBSD maps of transverse metallographic sections in AA7050 forged under different pre-aging deformation temperatures, illustrating how forging history modifies grain morphology and recrystallization-sensitive microstructure across the section. Adapted from Ref. [11] under CC BY 4.0. (**a**) Map A1; (**b**) Map A2; (**c**) Map A3, showing section-dependent grain morphology and orientation distribution under different pre-aging deformation temperatures.

**Figure 11:**
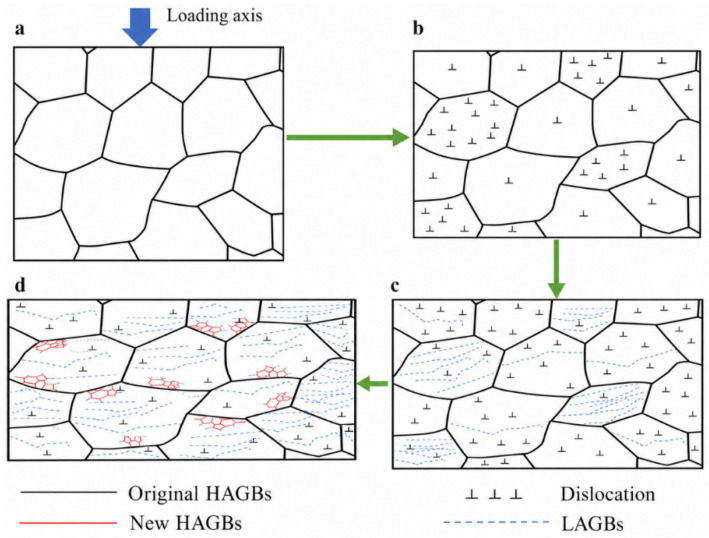
EBSD-based evolution of grain orientation, GND density, and boundary-state development in AA7050 during hot deformation, illustrating the continuous dynamic recrystallization pathway from undeformed grains to recrystallized grains bounded by high-angle grain boundaries [17]. Adapted from Ref. [17] under the publisher-declared Creative Commons license. (**a**) undeformed grains before loading; (**b**) dislocation accumulation during deformation; (**c**) formation of low-angle grain boundaries; (**d**) development of new high-angle grain boundaries during continuous dynamic recrystallization.

This point is not merely academic. In large forged aluminum components, the short-transverse direction often remains the most critical for ductility and fracture resistance, so the key question is how forging and subsequent heat treatment generate direction-dependent grain structures, textures, and precipitate populations through the section thickness. AA7050 and AA7075 studies have directly linked these features to anisotropic tensile behavior, fracture resistance, and metal-flow-controlled microstructural gradients. Figure 10 and Figure 11 present representative EBSD-based evidence for section-dependent grain architecture and continuous dynamic recrystallization in forged aluminum alloys.

### 5.3. Comparison Between 7xxx and Al-Li-Related Scale Coupling

The multiscale discussion becomes broader when Al-Li alloys are considered alongside 7xxx forgings. Although their strengthening routes differ, both material families show that aerospace forgings cannot be optimized by composition design alone. In 7xxx alloys, the dominant issues are the interaction among recrystallization state, texture, grain-boundary precipitation, and η-related strengthening sequences. In Al-Li alloys, the challenge shifts toward coupling deformation texture, second-phase fragmentation, Li-containing precipitates, and anisotropy reduction while preserving low density and high specific stiffness [21,22,23].

**Figure 12:**
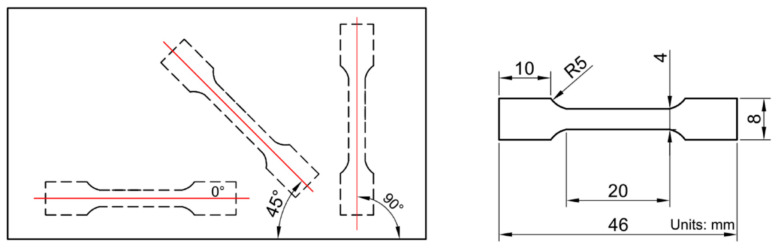
Representative microstructure–anisotropy comparison for an Al-Li alloy, illustrating how deformation path modifies texture development, second-phase distribution, and directional mechanical response [21]. Adapted from Ref. [21] under CC BY 4.0.

This comparison reinforces the central thesis of the manuscript: aerospace forging performance is governed by coupled structural features distributed across scales rather than by isolated average metrics. Figure 12 highlights how microstructure, texture development, and directional mechanical response remain interdependent in lightweight aluminum aerospace systems.

### 5.4. Environmental Degradation and Surface Protection in Aerospace Forgings

Environmental degradation should also be considered within the process–structure–property framework because surface condition, residual stress, grain-boundary chemistry, and precipitate distribution affect corrosion-assisted damage. In atmospheric service, chloride-containing moisture, sulfur- and nitrogen-containing pollutants, cyclic humidity, and temperature fluctuations can destabilize passive films or create occluded local chemistries. Titanium alloys usually rely on a stable TiO_2_ passive film but may suffer fretting-assisted oxidation or crevice attack in severe local environments. High-strength aluminum forgings are more susceptible to pitting, exfoliation, intergranular corrosion, and stress-corrosion cracking because precipitate-free zones, grain-boundary precipitates, and Cu/Zn/Mg-rich constituents create local electrochemical differences. Nickel-based superalloys mainly face oxidation and hot corrosion at elevated temperature, where protective chromia/alumina scales can be degraded by salts or combustion-derived contaminants [86,87,88,89].

Protection strategies should therefore be alloy- and surface-state-specific. Aluminum forgings commonly require solution/aging control to reduce grain-boundary susceptibility, surface sealing, anodizing or conversion coatings, corrosion-inhibiting primers, organic topcoats, and careful control of galvanic contact. Titanium components benefit from surface finishing, fretting-resistant coatings, and isolation from aggressive crevice environments. Nickel-based superalloy forgings may require oxidation-resistant coatings, diffusion aluminizing, MCrAlY-type bond coats, thermal-barrier systems, and periodic inspection of salt-affected regions. These protective measures are most effective when coupled with microstructural control and residual-stress management rather than applied as late-stage surface treatments alone [86,87,88,89].

## 6. From Characterization to Predictive Multiscale Modeling

### 6.1. Physics-Informed and Data-Enabled Strategies

A multiscale review becomes more useful when characterization outputs are converted into predictive capability rather than left as descriptive endpoints. For aerospace die forgings, phase constitution, grain topology, recrystallized fraction, boundary character, surface state, and precipitate descriptors should be treated as model inputs, calibration targets, or validation metrics for crystal-plasticity, phase-field, cellular-automata, and ICME-style frameworks [39,40,41,42,43,44,45,46,47,48,49,63].

**Figure 13:**
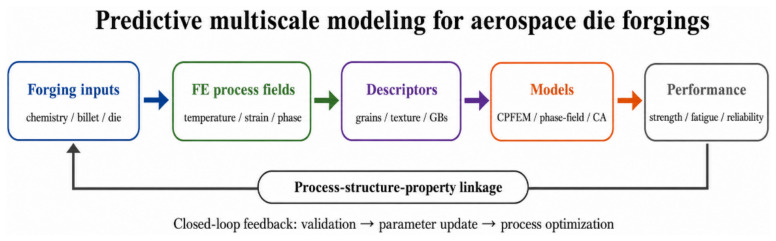
Author-created schematic of the process–structure–property modeling route for aerospace die forgings, showing how forging inputs, thermomechanical fields, microstructural descriptors, multiscale models, and performance prediction are integrated for closed-loop process optimization, based on concepts summarized in Refs. [39,40,41,42,43,44,45,46,47,48,49].

Several methodological routes are especially promising. Microstructure-sensitive crystal plasticity can transform measured orientation distributions, phase morphologies, and constituent-level properties into predictions of local strain partitioning and hotspot formation. Phase-field and related mesoscale approaches can represent phase morphology, grain growth, and recrystallization-sensitive structures under thermally and mechanically coupled conditions. Coupled FE-CA or FE-mesoscale frameworks provide a practical bridge from macroscale thermomechanical fields to local microstructure evolution, while ICME and data-enabled workflows increasingly connect chemistry, process history, and nanoscale descriptors to target properties. Figure 13 summarizes this process-structure-performance route.

### 6.2. Calibration Limits and Industrial Transfer

Predictive modeling for aerospace die forgings must remain grounded in real structural heterogeneity. Forged components often contain strong location-dependent gradients in strain, temperature, grain size, boundary state, precipitate distribution, surface condition, and defect inheritance; therefore, single-scale or spatially averaged calibration is rarely sufficient for robust industrial transfer. The most credible route is a closed loop in which macroscale process simulation identifies thermomechanical extremes, correlative characterization resolves the local structural state at critical locations, and reduced-order, phase-field, crystal-plasticity, or CA-based models explain the local response before feedback is returned to schedule design, die-path adjustment, or heat-treatment optimization.

This distinction separates visually attractive simulation from decision-capable modeling. A model that only reproduces a representative micrograph has limited industrial value, whereas a model that propagates experimentally measured heterogeneity through the hierarchy of process, structure, and response is more relevant to aerospace forging optimization. The dominant multiscale issues of the three principal alloy classes are compared in Table 2, which also highlights why future progress depends on combining physically meaningful descriptors with scalable computational workflows.

Risk-control solutions can be summarized as follows: titanium forgings require control of the α/β fraction through sub-transus or near-β processing, aging, prior-β grain refinement, and verification of dwell/fatigue-sensitive microtexture regions; nickel-based superalloys require billet-state homogenization, regulation of γ′/delta dissolution and reprecipitation, prevention of uncontrolled mixed grains, and validation of deliberately designed rim/bore gradients; high-strength aluminum forgings require optimized solution-aging treatment, control of η′/η and grain-boundary precipitation, reduction in recrystallization bands and short-transverse anisotropy, and integration of surface protection with fracture-toughness testing.

## 7. Conclusions and Outlook

Aerospace die forgings are intrinsically multiscale materials whose reliability emerges from coordinated control of phase constitution, grain architecture, recrystallization pathway, precipitate evolution, surface condition, and defect population across processing stages. Therefore, phase analysis, grain analysis, precipitation analysis, surface analysis, and defect assessment should not be treated as independent topics in review writing or process design. A major trend is the shift from qualitative micrograph comparison toward quantitative descriptor extraction, including grain-size distributions, boundary-character fractions, recrystallized fractions, KAM/GND metrics, precipitate size-density statistics, pore-size distributions, and surface/topography descriptors.

Across titanium alloys, wrought nickel-based superalloys, and high-strength aluminum forgings, the literature reveals a common principle: the most informative studies link thermomechanical variables to local structural state and then to property consequence. This logic is equally important for figure selection, correlative characterization, and predictive modeling. A second trend is the increasing use of correlative workflows in which EBSD/3D-EBSD, TEM/STEM, APT, tomography, diffraction, residual-stress measurements, environmental-degradation assessment, and local mechanical testing are combined to connect the same processing history to multiple descriptors across scales. This development is important because no single technique can explain phase stability, grain architecture, precipitation, defect inheritance, environmental sensitivity, and mechanical response at the same time.

The immediate priorities are greater use of correlative and three-dimensional characterization, stronger extraction of quantitative descriptors, better treatment of uncertainty and location dependence, and tighter integration between experiment and microstructure-sensitive modeling. A third trend is the transition from descriptive characterization to predictive, uncertainty-aware modeling. Crystal plasticity, phase-field modeling, cellular automata, FE-mesoscale coupling, surrogate modeling, and ICME workflows are increasingly used to convert measured descriptors into process-window design, local-property prediction, and closed-loop optimization. Future work should focus on validated three-dimensional datasets, standardized descriptor definitions, scale-aware uncertainty quantification, environmental-degradation coupling, and industrially transferable models that can guide die design, forging schedule selection, heat-treatment optimization, inspection planning, and surface-protection strategies.

## Figures and Tables

**Table 1:** Representative multiscale analysis targets for aerospace die forgings.

Scale/Domain	Main Scientific Question	Representative Methods (Scale-Overlapping, Not Exclusive)	Typical Outputs
Macro	Did forging, heat treatment, and metal flow create bulk integrity and acceptable gradients?	Macro-etching; ultrasonic inspection; X-ray/CT; residual-stress mapping; mechanical testing	Flow lines; dimensional integrity; internal soundness; bulk anisotropy
Meso	Where are strain, temperature, and texture gradients concentrated?	Sectioned EBSD maps; local hardness; mesoscale tomography; laboratory/synchrotron X-ray CT; process simulation	Localization bands; surface/core gradients; regional heterogeneity
Micro	How do grains, boundaries, recrystallized regions, and textures evolve?	EBSD; OM/SEM; parent-phase reconstruction; micro-XRD/synchrotron diffraction; X-ray microscopy	Grain-size distribution; HAGB/LAGB fraction; texture; KAM; recrystallized fraction
Sub-micro	How do dislocations, subgrains, and interfaces change during forging?	TEM/STEM; HR-EBSD; ECCI	Substructure; slip traces; local strain concentration; boundary mobility
Nano/atomic	What is the precipitation and segregation state?	APT; high-resolution STEM; nanoscale diffraction/spectroscopy; atomistic chemical mapping	Precipitate chemistry; interfacial segregation; strengthening-state metrics

Note: The methods listed in Table are representative rather than scale-exclusive. For example, X-ray-based methods range from laboratory radiography/CT for bulk integrity to synchrotron diffraction, micro-XRD, X-ray microscopy, and high-resolution CT for microstructural or defect-scale characterization. The appropriate scale should therefore be defined by spatial resolution, sampling volume, and the target descriptor rather than by the instrument name alone.

**Table 2:** Main multiscale issues in the three principal aerospace forging material classes. Risk-control solutions are summarized immediately below the table to avoid treating the listed risks as unresolved problems.

Material Class	Dominant Phase Questions	Dominant Grain/Texture Questions	Dominant Property-Risk Linkage
Titanium die forgings	α/β fraction; prior β reconstruction; globularization vs. transformed β morphology	β-grain refinement; recrystallized β microtexture; variant selection in α precipitation	Fatigue/dwell sensitivity; notch sensitivity; strength–ductility balance
Ni-based superalloy die forgings	γ′ dissolution/reprecipitation; δ-phase control; carbide/secondary-phase stability	Dynamic/post-dynamic recrystallization; boundary pinning; grain-boundary character	Creep/fatigue resistance; grain-growth instability; local property scatter
High-strength Al die forgings	GP zones; η′/η evolution; dispersoid and grain-boundary precipitation control	Partially recrystallized structure; fiber texture; CDRX and anisotropy control	Short-transverse ductility loss; toughness anisotropy; over-aging/strength tradeoff

## Data Availability

No new data were created or analyzed in this review. Data sharing is not applicable to this article.
